# Artificial intelligence in acupuncture: bridging traditional knowledge and precision integrative medicine

**DOI:** 10.3389/fmed.2025.1633416

**Published:** 2025-07-31

**Authors:** Guo-Liang Hou, Bao-Qiang Dong, Ben-Xing Yu, Jian-Yu Dai, Xing-Xing Lin, Ze-Zhong Cheng

**Affiliations:** School of Acupuncture and Massage, Liaoning University of Traditional Chinese Medicine, Shenyang, Liaoning, China

**Keywords:** acupuncture, artificial intelligence, deep learning, natural language processing, personalized medicine, outcome prediction

## Abstract

The integration of artificial intelligence (AI) into acupuncture research is accelerating the transformation of this traditional, experience-based practice into a data-driven, precision discipline. This review synthesizes recent advances in AI-enabled outcome prediction techniques, encompassing deep learning, meta-analytic modeling, natural language processing (NLP), computer vision, and neuroimaging-based analysis. For instance, convolutional neural networks (CNNs) have been successfully applied to classify tongue images and detect ZHENG patterns, while transformer-based NLP models enable automated extraction of clinical knowledge from classical texts. These technologies improve diagnostic objectivity, standardize treatment planning, and facilitate individualized care by enabling longitudinal efficacy modeling and real-time monitoring. Despite their potential, current implementations are constrained by limited and heterogeneous datasets, annotation variability, and gaps in clinical validation. We analyze key methodological innovations and challenges, and recommend future directions including the construction of federated multimodal data platforms, development of explainable AI frameworks, and promotion of open science practices. This convergence of AI and acupuncture presents a unique opportunity to enhance scientific rigor, clinical utility, and global integration of acupuncture within the paradigm of precision integrative medicine.

## Introduction

Acupuncture has long been regarded as a foundational component of traditional East Asian medicine, with widespread clinical application in managing chronic pain, neurological conditions, and functional syndromes. Its holistic approach and individualized treatment philosophy have contributed to its growing global popularity. Nonetheless, the integration of acupuncture into modern biomedical systems remains limited. A key barrier lies in the reliance on subjective diagnostic techniques (1)—such as tongue inspection and pulse palpation—which vary considerably across practitioners and lack standardized evaluation protocols (2, 3). In recent years, the advent of artificial intelligence (AI) has introduced transformative opportunities for addressing these challenges. By enabling automated analysis of high-dimensional and multimodal clinical data, AI methods such as deep learning and natural language processing (NLP) can support more objective, reproducible, and scalable approaches to diagnosis, treatment planning, and outcome prediction (4–6). For instance, convolutional neural networks (CNNs) have demonstrated potential in classifying visual features from tongue images, while transformer-based NLP models can extract structured clinical knowledge from unstructured texts. Moreover, AI-driven predictive modeling has opened new pathways for personalizing acupuncture interventions. Machine learning algorithms, when trained on large datasets, are capable of forecasting therapeutic response trajectories and identifying patient subgroups most likely to benefit from specific acupoint protocols (3, 7–9). At the same time, tools such as computer vision and real-time monitoring systems have been developed to enhance procedural safety and consistency during needling. Despite these promising developments, several critical gaps remain. Data limitations—including small sample sizes, inconsistent labeling standards, and heterogeneity in study design—continue to hinder generalizability. The opaque nature of many AI models also raises concerns about interpretability and clinical trustworthiness (10, 11). In addition, there is a pressing need for open-access infrastructure and collaborative platforms to promote data sharing and reproducible research in this emerging field.

This review seeks to synthesize recent advances in AI-enabled acupuncture outcome prediction, with a focus on methodological innovation, clinical application, and future development pathways. By bridging traditional therapeutic knowledge with modern computational techniques, we aim to contribute to the evolving field of precision integrative medicine and highlight how AI may facilitate a more rigorous, effective, and globally accepted form of acupuncture.

This table summarizes representative AI approaches applied in acupuncture-related studies, the typical input data used, their clinical roles, and corresponding implications for personalized medicine and decision support.

## Data-driven insights and predictive strategies in acupuncture

**FIGURE 1:**
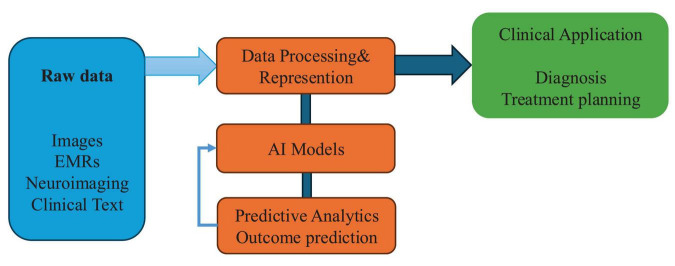
Conceptual framework of artificial intelligence (AI) integration in acupuncture research. Conceptual framework illustrating how artificial intelligence integrates with key components of acupuncture research—from raw data processing to predictive modeling and clinical application. This model reflects the data-driven pipeline toward precision acupuncture.

Recent advances in deep learning technologies—particularly convolutional neural networks (CNNs)—have significantly enhanced the objectivity and accuracy of acupuncture outcome prediction by enabling the extraction of subtle, clinically relevant visual biomarkers from medical images. Traditional assessments of diagnostic features such as tongue morphology, pulse condition, and facial complexion have long depended on practitioner experience, introducing considerable subjectivity and inter-observer variability (12, 13). CNNs provide a powerful alternative, capable of automatically identifying intricate patterns in visual data that often exceed human observational capability (14, 15). For example, Han et al. employed the SEResNet101 architecture to analyze tongue images as non-invasive biomarkers for predicting subthreshold depression and acupuncture responsiveness. The model demonstrated high predictive accuracy and diagnostic reliability, offering a quantitative and reproducible approach to clinical assessment. Notably, the incorporation of attention mechanisms within CNNs has further improved model performance by enabling selective focus on diagnostically relevant image regions—such as localized changes in tongue texture, color gradients, and anatomical irregularities indicative of pathological states (2). These deep learning frameworks represent a major step toward standardizing diagnostic evaluations in acupuncture. By transitioning from subjective interpretation to algorithmically driven assessments, they help reduce practitioner bias and improve inter-rater consistency (15). Importantly, such innovations not only establish new benchmarks for assessing acupuncture efficacy but also support the integration of acupuncture into evidence-based clinical workflows. The enhanced objectivity and scalability of deep learning models offer strong potential for their adoption in routine practice and their incorporation into broader precision medicine strategies (16, 17). This Al-driven workflow is conceptually illustrated in Figure 1.

Model-based longitudinal meta-analysis has emerged as a critical tool for characterizing the temporal dynamics and efficacy of acupuncture interventions, particularly in chronic pain management. Conventional acupuncture studies often rely on short-term outcomes or isolated time points, limiting their ability to reveal treatment trajectories or account for inter-individual variability (18). To address this gap, Li et al. applied non-linear mixed-effects modeling to aggregate data from randomized controlled trials (RCTs), demonstrating that cumulative treatment duration is strongly associated with clinically meaningful analgesic outcomes. These longitudinal models offer distinct advantages. By capturing time-dependent response patterns and integrating trial-level covariates—such as baseline pain severity, pain localization, and demographic characteristics—they enable more nuanced evaluations of treatment efficacy (18). Importantly, the quantitative insights derived from meta-analytic modeling provide a foundation for integrating machine learning algorithms trained on individual patient-level data. This creates opportunities for personalized prediction of acupuncture effectiveness, optimizing treatment frequency, duration, and acupoint combinations for specific patient subgroups (3). In this way, longitudinal meta-analytic frameworks contribute not only to methodological rigor and reproducibility but also to the development of clinically actionable, evidence-based treatment strategies in modern acupuncture research.

Acupoint selection in traditional acupuncture has historically relied on empirical knowledge derived from classical texts and practitioner experience, often resulting in variability across clinical practices. Recent developments in data mining techniques offer a systematic, evidence-based approach to identifying effective acupoint combinations, bridging traditional wisdom with modern analytical rigor (5). For instance, Hwang et al. applied association rule mining—specifically the *A priori* algorithm—to large-scale datasets from randomized controlled trials on pain management. Their analysis revealed statistically significant and frequently co-occurring acupoint pairs and clusters, such as SP6 (Sanyinjiao), ST36 (Zusanli), LI4 (Hegu), and LR3 (Taichong), which consistently demonstrated therapeutic efficacy (5, 19). Beyond validation, data mining enables the discovery of hidden relationships and synergistic patterns among acupoints that may not be apparent through clinical experience alone (20). By uncovering these complex interdependencies, researchers can construct intelligent decision-support systems that recommend personalized acupoint prescriptions based on patient profiles, symptom patterns, and historical treatment data (21). These systems enhance treatment precision, reduce practitioner variability, and pave the way for scalable, reproducible acupuncture protocols aligned with the principles of precision medicine. A summary of representative AI techniques and their clinical acupuncture applications is presented in Table 1.

**TABLE 1:** Mapping of artificial intelligence (AI) techniques to clinical acupuncture applications.

AI technique	Input modalities	Clinical functions	Application relevance
Convolutional neural networks (CNNs)	Tongue images, facial photos, pulse waveforms	Syndrome differentiation, disease pattern recognition	Automated diagnostic assistance
Transformer-based language models	Clinical records, ancient texts, EMRs	Knowledge extraction, intelligent decision support	Building explainable acupuncture knowledge bases
Recurrent neural networks (RNNs)	Longitudinal symptom scores, time-series outcomes	Time-series outcome prediction, therapy responsiveness	Personalized dynamic response forecasting
Graph neural networks (GNNs)	Acupoint co-occurrence matrices, point-symptom networks	Acupoint synergy modeling, functional connectivity mapping	Network-based acupuncture planning
Clustering algorithms	Patient symptoms, demographic profiles, ZHENG labels	Patient stratification, subtype discovery	Precision treatment targeting by subtypes
Support vector machines (SVMs)	Baseline clinical features, imaging parameters	Binary classification (responder vs. non-responder)	Outcome prediction for clinical decision-making
Ensemble learning models	Integrated datasets	Multimodal prediction and treatment optimization	Holistic and integrative precision medicine strategies

Neuroimaging biomarkers have emerged as valuable resources for predicting acupuncture treatment outcomes, particularly in neurological and psychiatric disorders. Magnetic resonance imaging (MRI), for example, can reveal structural and functional brain alterations associated with therapeutic response. Yang et al. utilized baseline gray matter volumes from MRI scans to distinguish responders from non-responders among migraine patients. By applying support vector machine (SVM) classifiers, the study achieved high predictive accuracy (22). The integration of feature selection techniques, such as the least absolute shrinkage and selection operator (LASSO), allowed for identification of key neuroanatomical predictors—including regions within the frontal, temporal, and parietal lobes. Moreover, observed neuroplastic changes following acupuncture provided mechanistic insight into brain adaptation during treatment. These findings demonstrate that neuroimaging-based predictive models can not only forecast clinical efficacy but also contribute to our understanding of acupuncture’s underlying biological mechanisms (23, 24).

Artificial intelligence techniques have also shown promise in predicting acupuncture efficacy across a broad range of functional disorders. For instance, in the case of functional dyspepsia (FD), Yin et al. applied SVM models to routine clinical data—including demographic features and baseline symptom scores—to predict both short-term and long-term outcomes of acupuncture treatment. The model achieved high predictive performance for both symptom improvement and quality-of-life metrics at 12 weeks follow-up (25). Notably, the study demonstrated that machine learning could generate robust predictions even in the absence of complex biomarkers like neuroimaging, highlighting the practicality of AI-based prediction models in real-world clinical settings. These findings reflect a broader shift toward predictive, preventive, personalized, and participatory (P4) medicine (26). By enabling individualized treatment planning based on accessible clinical information, AI-driven tools can extend the benefits of precision medicine into the realm of complementary and integrative healthcare (27).

## Technological applications enhancing acupuncture practice

To provide a structured overview of artificial intelligence techniques applied in clinical acupuncture, Table 1 summarizes representative AI approaches, their data modalities, and key clinical applications. Each subsequent section explores these techniques and their implications for clinical practice in greater detail. Advancements in natural language processing (NLP) have substantially enhanced the ability to extract, interpret, and structure acupuncture-related information from vast repositories of biomedical texts. Traditional acupuncture literature contains rich clinical knowledge and anatomical insights, but manual extraction of relevant content is labor-intensive and prone to subjectivity. Leveraging transformer-based language models such as GPT-3.5 and GPT-4, researchers have made notable progress in automating relation extraction tasks tailored to acupuncture. Li et al. demonstrated that fine-tuned large language models (LLMs) outperform conventional deep learning approaches—such as long short-term memory (LSTM) networks and domain-specific tools like BioBERT—in identifying anatomical and spatial relationships between meridians, acupoints, and therapeutic indications (28). Transformer-based architectures excel in handling long-range dependencies and contextual nuances, enabling accurate interpretation of complex relational data across multiple sentences. By facilitating the construction of machine-interpretable acupuncture knowledge bases, these NLP systems support the development of intelligent decision-support tools. Such systems can offer evidence-based recommendations for acupoint selection, treatment planning, and individualized therapy, thereby advancing the field toward precision acupuncture (29, 30). Ultimately, NLP integration serves as a critical bridge between traditional textual knowledge and modern computational analytics. However, transformer-based models face notable limitations when applied to acupuncture and TCM literature, including domain mismatch with Western biomedical pre-training corpora, a lack of annotated datasets, and potential hallucination of unsupported facts, which may compromise reliability in clinical applications.

In parallel, artificial intelligence–powered computer vision technologies are increasingly being used to enhance procedural safety and monitoring during acupuncture treatment. Traditionally, the accuracy of needle placement has depended on practitioner skill and real-time judgment, which introduces variability and risk. To address this, Lin et al. developed an automated needle detection system using the YOLOv8 deep learning framework (9). The system applies advanced image preprocessing techniques—such as cropping, resizing, and augmentation—to ensure robust detection under varied clinical conditions, including low contrast and occlusion. It achieves high real-time accuracy in identifying acupuncture needles, even in challenging environments. By integrating automated alerts, the system supports consistent adherence to safety protocols and reduces the likelihood of errors such as needle misplacement, breakage, or retention. This approach exemplifies the broader trend of embedding real-time, standardized monitoring technologies into traditional acupuncture workflows. These innovations promote procedural reliability, mitigate practitioner-dependent variability, and contribute to the overall safety and quality assurance of acupuncture practice.

## Challenges and future directions in ai-enhanced acupuncture

Despite the substantial progress enabled by data-driven and AI-based approaches in acupuncture outcome prediction, several methodological and technical challenges continue to limit their clinical applicability and generalizability. A key issue lies in the limited size and heterogeneity of existing datasets. Many current predictive models are trained on small, homogeneous cohorts, which undermines their external validity across diverse clinical populations and settings (17). Particularly in imaging and neuroimaging studies, models often exhibit strong performance within narrowly defined datasets but suffer from reduced accuracy when applied to real-world, heterogeneous scenarios—a phenomenon known as overfitting (31). This limitation underscores the urgent need for large-scale, multicenter datasets that reflect the variability of clinical practice and patient characteristics in acupuncture. Another critical barrier involves the annotation and labeling of training data, especially for imaging and textual modalities. Manual annotation is not only time-consuming and resource-intensive but also prone to inconsistencies due to inter-observer variability (32). Differences in clinical expertise, interpretation of anatomical features, and subjective judgments during data labeling can introduce substantial bias into AI training pipelines (33). These inconsistencies reduce the robustness and reproducibility of predictive models and present significant obstacles for broader clinical adoption. In the context of NLP, the scarcity of domain-specific annotated corpora remains a major challenge. Although transformer-based models like GPT have demonstrated promising capabilities in relation extraction and biomedical comprehension, their performance in acupuncture-specific applications is limited by the lack of high-quality, standardized textual datasets (34). Acupuncture texts often contain complex, ambiguous, or non-standardized terminologies, which further complicates automated information extraction and downstream clinical integration.

For neuroimaging-based predictive modeling, technical variability introduces another layer of complexity. Differences in imaging hardware, scanning protocols, and data preprocessing pipelines across institutions may introduce artifacts or confounding factors that compromise model generalizability (35). Furthermore, brain structure and function are inherently dynamic and individualized, influenced by a range of factors including age, pathology, and treatment response. These physiological variabilities challenge the stability and reproducibility of neuroimaging biomarkers in acupuncture research. Taken together, these limitations highlight the need for standardized data collection protocols, inter-institutional collaboration, and open-access data sharing to promote model reproducibility and scalability (36). Establishing comprehensive, annotated, and interoperable datasets—spanning clinical records, imaging data, and textual corpora—will be essential to ensure that AI-powered tools can transition from experimental validation to routine clinical deployment. Without addressing these foundational barriers, the full potential of AI-driven precision acupuncture may remain unrealized.

The advancement of AI-driven predictive modeling in acupuncture presents a transformative opportunity for personalized, evidence-based integrative medicine. However, realizing this potential requires addressing current methodological limitations and actively shaping future development across multiple domains. One priority lies in building large-scale, multimodal, and interoperable datasets that integrate clinical records, neuroimaging scans, patient-reported outcomes, genetic and epigenetic profiles, and demographic data (37). These datasets enable the application of advanced machine learning architectures—including convolutional neural networks (CNNs), recurrent neural networks (RNNs), and transformer-based models—to analyze complex clinical information holistically (38). To ensure data consistency and replicability across institutions, standardized data frameworks such as the Omaha System should be widely adopted (39). Promising examples include the China Acupuncture Clinical Data Alliance (CACDA), which has launched a nationwide network to collect and standardize acupuncture-related data, and the Open Health AI Consortium, which promotes federated learning approaches for secure data collaboration. Other global initiatives, such as the Observational Health Data Sciences and Informatics (OHDSI) program and the Global Alliance for Genomics and Health (GA4GH), provide scalable models for harmonizing diverse datasets and promoting interoperability in biomedical research (40, 41). Improving transparency is also essential for clinical adoption. Explainable AI (XAI) techniques—such as SHAP, LIME, and attention-based visualization—can illuminate how specific features (e.g., symptom profiles, neuroimaging patterns, or acupoint usage) influence model predictions (42, 43). In parallel, adaptive learning systems that incorporate real-world clinical feedback and newly acquired data will allow for continuous model refinement and sustained relevance (44). Transformer-based NLP models have the potential to revolutionize acupuncture decision support. Future developments should leverage domain-specific ontologies and annotated corpora to improve model performance in tasks such as acupoint relation extraction, symptom clustering, and treatment recommendation generation (28, 45). These systems could integrate clinical histories, trial outcomes, and classical acupuncture literature to generate context-aware, personalized recommendations and reduce clinical variability. In terms of procedural safety, real-time sensing systems are emerging as important adjuncts to AI-enhanced acupuncture. Expanding upon needle detection systems like YOLOv8, future platforms could integrate biosensors, computer vision, and real-time video analytics to monitor patient status and needle precision during treatment (9, 46). Such systems would provide immediate feedback and ensure adherence to standardized protocols, particularly in high-throughput or educational settings. A particularly promising area involves integrating multi-omics data—such as genomic, epigenomic, proteomic, and metabolomic profiles—into AI-driven acupuncture models. These biological layers may help identify responder subtypes and inform precision treatment strategies for conditions like chronic pain, insomnia, and gastrointestinal disorders (47). This approach enables personalized planning of acupoint combinations, stimulation intensity, and treatment frequency according to molecular and physiological profiles (3).

Lastly, the scalability and sustainability of AI in acupuncture research will depend on shared standards and open science practices. Unified protocols for study design, data annotation, and result reporting are needed to ensure replicability across centers (45, 48). Open-access data repositories and federated learning frameworks will facilitate privacy-preserving data sharing and model validation at scale (49). Furthermore, interdisciplinary collaboration—among clinicians, AI researchers, bioinformaticians, and policy experts—will be critical to accelerate translation and reinforce acupuncture’s credibility within evidence-based medicine (50). By addressing these foundational and future-facing considerations, the field of AI-enhanced acupuncture can advance toward clinically robust, transparent, and individualized applications that align with global efforts in precision and integrative healthcare.

## Discussion

The integration of artificial intelligence (AI) and data-driven methodologies into acupuncture research represents a paradigm shift from traditional experiential practice toward standardized, objective, and personalized care. Historically, the assessment of acupuncture efficacy has relied heavily on practitioner expertise, subjective clinical judgment, and patient-reported outcomes, which limits reproducibility, comparability, and broader clinical integration. Recent advancements in deep learning, natural language processing (NLP), computer vision, and meta-analytic modeling have fundamentally reshaped this landscape by introducing quantitative rigor, automation, and predictive precision.

Deep learning approaches—particularly convolutional neural networks (CNNs)—have greatly enhanced the objectivity and accuracy of acupuncture diagnostics. These models are capable of identifying subtle clinical features from visual inputs such as tongue images or facial patterns, often surpassing the diagnostic reliability of human practitioners (6, 9). The inclusion of attention mechanisms allows these models to focus on diagnostically significant image regions, further improving performance. As a result, CNNs provide a scalable foundation for standardizing diagnosis and monitoring in clinical acupuncture practice (28). Similarly, model-based longitudinal meta-analyses offer a statistically robust framework for evaluating the time-dependent efficacy of acupuncture interventions. These models can synthesize heterogeneous clinical trials, account for inter-patient variability, and identify key factors such as treatment duration, symptom severity, and demographic modifiers that influence therapeutic response (50). By transforming aggregate trial data into dynamic response trajectories, they create a quantitative foundation upon which machine learning algorithms can be trained and validated. NLP technologies have also shown transformative potential in structuring unstructured clinical knowledge. By leveraging large language models (LLMs) such as GPT, researchers can now extract and organize complex semantic relationships among acupoints, meridians, anatomical landmarks, and treatment principles from vast biomedical corpora (49, 50). These structured knowledge representations facilitate the development of intelligent decision-support tools that provide real-time, evidence-based recommendations for individualized treatment planning (51). Real-time computer vision applications, such as YOLOv8-based needle tracking systems, offer a practical solution to long-standing safety concerns in acupuncture procedures. These AI-driven platforms continuously monitor needle insertion and positioning, reducing human error and ensuring procedural consistency across practitioners of varying experience levels (52). Such systems exemplify the potential of embedding digital safety infrastructure into traditional therapeutic workflows (53). Despite these notable advancements, several barriers remain. Chief among them is the limited generalizability of predictive models due to small sample sizes, data heterogeneity, and annotation bias in current datasets (54). Overfitting remains a major concern, particularly in neuroimaging and image-based models, where data scarcity and institutional variability compromise robustness. Manual data labeling, often subject to inter-observer variability, further constrains reproducibility and scalability (24).

Integrating multimodal datasets—such as neuroimaging, clinical records, and textual information—poses additional methodological complexity. These datasets differ not only in format and scale but also in the temporal and semantic granularity of the information they contain. Developing reliable data fusion strategies that preserve signal integrity across modalities remains an ongoing challenge (2, 6). Additionally, acupuncture’s inherent variability in technique, acupoint prescription, and patient response complicates the development of universally applicable models, necessitating stronger efforts toward standardization. Addressing these challenges will require coordinated, interdisciplinary collaboration across clinicians, data scientists, bioinformaticians, and regulatory stakeholders. Future priorities include building large, diverse, and well-annotated datasets; implementing explainable and adaptive AI models; and fostering open science practices to enable reproducible validation and deployment. Bridging methodological innovation with clinical translation is essential to embedding AI into everyday acupuncture care and realizing the broader vision of precision integrative medicine (52, 55).

## Conclusion

The integration of artificial intelligence (AI) and data-driven methodologies into acupuncture research signifies a transformative shift from traditional empirical practices toward standardized, objective, and personalized care. Advances in deep learning, natural language processing, computer vision, and meta-analytic modeling have enabled precise identification of clinical biomarkers, reproducible outcome prediction, and improved procedural safety, thereby enhancing the scientific credibility and clinical applicability of acupuncture. However, the full potential of AI in this domain remains constrained by challenges such as limited data diversity, inconsistent clinical protocols, annotation variability, and the lack of standardized infrastructure. To address these limitations and realize scalable implementation, future efforts must prioritize the development of interoperable, multimodal datasets, adoption of explainable and adaptive AI models, and promotion of interdisciplinary collaboration. By embedding genomic, neuroimaging, and patient-reported data into predictive frameworks and fostering transparency in data sharing and methodology, the field is poised to advance toward truly personalized acupuncture care within the broader landscape of precision and integrative medicine.
